# The NTD Nanoscope: potential applications and implementations

**DOI:** 10.1186/1471-2105-12-S10-S21

**Published:** 2011-10-18

**Authors:** Stephen Winters-Hilt, Evenie Horton-Chao, Eric Morales

**Affiliations:** 1 Dept. of Computer Science, University of New Orleans, 2000 Lakeshore Drive, New Orleans, LA 70148, USA; 2 Meta Logos Incorporated, 6218 Waldo Dr., New Orleans, LA 70122, USA

## Abstract

**Background:**

Nanopore transduction detection (NTD) offers prospects for a number of highly sensitive and discriminative applications, including: (i) single nucleotide polymorphism (SNP) detection; (ii) targeted DNA re-sequencing; (iii) protein isoform assaying; and (iv) biosensing via antibody or aptamer coupled molecules. Nanopore event transduction involves single-molecule biophysics, engineered information flows, and nanopore cheminformatics. The NTD Nanoscope has seen limited use in the scientific community, however, due to lack of information about potential applications, and lack of availability for the device itself. Meta Logos Inc. is developing both pre-packaged device platforms and component-level (unassembled) kit platforms (the latter described here). In both cases a lipid bi-layer workstation is first established, then augmentations and operational protocols are provided to have a nanopore transduction detector. In this paper we provide an overview of the NTD Nanoscope applications and implementations. The NTD Nanoscope Kit, in particular, is a component-level reproduction of the standard NTD device used in previous research papers.

**Results:**

The NTD Nanoscope method is shown to functionalize a single nanopore with a channel current modulator that is designed to transduce events, such as binding to a specific target. To expedite set-up in new lab settings, the calibration and troubleshooting for the NTD Nanoscope kit components and signal processing software, the NTD Nanoscope Kit, is designed to include a set of test buffers and control molecules based on experiments described in previous NTD papers (the model systems briefly described in what follows). The description of the Server-interfacing for advanced signal processing support is also briefly mentioned.

**Conclusions:**

SNP assaying, SNP discovery, DNA sequencing and RNA-seq methods are typically limited by the accuracy of the error rate of the enzymes involved, such as methods involving the polymerase chain reaction (PCR) enzyme. The NTD Nanoscope offers a means to obtain higher accuracy as it is a single-molecule method that does not inherently involve use of enzymes, using a functionalized nanopore instead.

## Introduction

The NTD Nanoscope offers the means to critically complete the SNP and RNA-seq data processing pipeline. Current methods used for DNA sequencing have error rates of approximately 1/1000 [1-4]. To take full advantage of the individualized medicine prospects, an error rate less than 1/100,000 is needed (this is one of the conditions to obtain the Archon X Prize for Genomics [5]). The 1/1000 error rate limitation is partly due to the enzymes used in the methods themselves having error rates of approximately 1/1000. DNA sequencing is fast becoming incredibly *in*expensive, however, disease conditions rarer than 1/1000 can be overlooked or misdiagnosed, and many genetic diseases are rarer than this. What is needed is targeted re-sequencing and SNP discovery in the important parts of the genome to have reduced error rates, and to do so in an inexpensive way.

Other nanoscopes typically have channel geometries with ~500 nm diameter cross-sections. The nanoscope we speak of here is meant to strongly interact at the single-molecule level, so we require channel geometries with diameter on the order of 1 nm. In the NTD functionalization the transducer molecule is drawn into the channel by an applied potential but is too big to translocate, instead becoming stuck in a bistable capture such that it modulates the channel’s ion-flow with stationary statistics in a distinctive way. If the channel modulator is bifunctional in that one end is meant to be captured and modulate while the other end is linked to an aptamer or antibody or ssDNA for specific binding (or annealing), then we have the basis for a remarkably sensitive and specific event transducer.

The NTD Nanoscope offers a means to do sensing with better than 1/1000 accuracy on any SNP variant or antigen. In [6], and the previous work cited there, it is shown that an error rate less than 1/1000 accuracy is possible in discerning DNA hairpins that differ only in a single base-pair in their stem regions. The 1/1000 error rate was obtained under conditions where only the first 100 ms of signal was used in the analysis. When using a DNA-hairpin modulator that has an attached interaction moiety, the signal change is much more pronounced, as is shown in [6], with error rates of less than 1/10,000 based on 200 ms signal samples. Thus, given the signal processing advantages that can be engineered into the transducer molecule, the detector sensitivity and specificity are only limited by the sensitivity and specificity of the interaction moiety for its target, and by the time of observation. The NTD Nanoscope, similarly, directly establishes a general-target (non-DNA) biosensor with sensitivity and specificity limited by that of the aptamer or antibody involved.

Other nanopore detector approaches use a fundamentally different experimental approach, one that involves polymer translocation and the blockade dwell-time observed for a translocation. There is a large literature based on this approach (see [7-30] for some of the most recent), but no commercially available nanopore detector is known at this time. A common feature employed in the translocation/dwell-time discrimination approaches is the blockade dwell-time, where the dwell-time is typically engineered to be associated with the lifetime until a specific bond failure occurs. Other dwell-time feature variations include time *until* a bond-formation occurs, or simply measuring the approximate length of a polymer according to its translocation dwell-time. For translocation-based approaches, blockade ‘level’ is usually a fixed level, and often not stationary, especially if one is trying to elicit non-stationary sequence information from the stationary blockade – whereas NTD tracks the non-stationary sequence information via corresponding phases of stationary statistics.

**Fig. 1:**
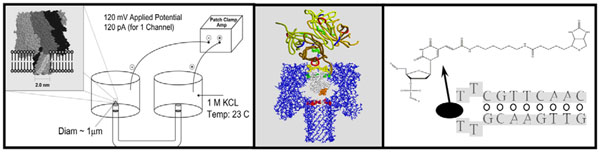
**Schematic diagram of the nanopore transduction detector.** The nanopore detector consists of a single pore in a lipid bilayer that is created by the oligomerization of the staphylococcal alpha-hemolysin toxin, and a patch clamp amplifier capable of measuring pico Ampere channel currents.

Transduction methods introduce different states to the channel via observations of changes in blockade statistics on a single molecular blockade event that is modulatory. This is an arrangement involving a partially-captured, single-molecule, channel modulator, typically with a binding moiety for a specific target of interest linked to the modulator’s extra-channel portion (see Fig. 1). The modulator’s state changes according to whether its binding moiety is bound or unbound. For a comparative analysis of the translocation and transduction approaches, see Table 1 below (from [6]).

**Table 1:** **Comparative analysis** of the Translocation/Dwell-Time (T/TD) approach and the Nanopore Transduction Detection (NTD) approach.

**(1) Feature Space.** The T/DT approach typically has a single feature, the dwell time. Sometimes a second feature, the fixed blockade level observed, is also considered, but usually not more features sought (or engineered) than that. The NTD approach has multiple features, e.g., blockade HMM parameters, etc., with number and type according to modulator design objectives.
**(2) Versatility.** T/DT: highly engineered/pre-processed for detection application to a particular target. NTD: requires minimal preparation/augmentation to the transduction platform via use of separately provided binding moieties (antibody or aptamer, for example) for particular target or biomarker (which are then simply linked to modulator)

**(3) Speed.** T/DT: Slow: entire detection “process” is at the channel, and typically restricted on processing speed to the average time-scale feature (dwell-time) for the longest-lived blockade signal class. NTD: Fast: feature extraction not dependent on dwell-time. Very low probability to get a false positive.

**(4) Multichannel.** T/DT: Method not amenable to multichannel gain with single-potential platform (can’t resolve single-channel blockade signal with multichannel noise). NTD: Have multichannel gain due to rich signal resolution capabilities of an engineered modulator molecule.

**(5) Feature Refinement/Engineering.** T/DT: No buffer modifications or off-channel detection extensions via introduction of substrates; the weak feature set limited to dwell-time doesn’t allow such methods to be utilized. NTD: Have “lock-and-key” level signal resolution. The introduction of off-channel substrates in the buffer solution can increase sensitivity.

**(6) Multiplex capabilities.** T/DT: Each modified channel is limited to detect a single analyte or single bond-change-event detection, so no multiplexing without brute force production of arrays of T/DT detectors in a semiconductor production setting. NTD: Supports multi-transducer, multi-analyte detection from a single sample. Supports multichannel with a single aperture.

The nanopore transduction detection platform (Fig. 1) involves functionalizing a nanopore detector platform in a new way that is cognizant of signal processing and machine learning capabilities and advantages, such that a highly sensitive biosensing capability is achieved [6]. The core idea in the NTD functionalization of the nanopore detector is to design a molecule that can be drawn into the channel (by an applied potential) but will be too big to translocate, instead becoming stuck in a bistable capture such that it modulates the ion-flow in a distinctive way. An approximately two-state telegraph signal has been engineered for a number of NTD modulators. Obtaining an NTD modulator begins with choosing a single molecule that with have a tight fit in a channel. For alpha-hemolysin nanopore detectors, this has been specifically engineered by tuning over a selection of DNA hairpin ‘gauges’[38] to arrive at a nine base-pair DNA hairpin, with a 4 dT loop, that possesses the geometric and charge properties to act as an NTD modulator, where an antibody linked to the 4 dT loop is examined in [36].

The use of a channel modulator introduces significant engineered signal analysis complexity that we resolve using artificial intelligence (machine learning) methods. The benefit of this complication is a significant gain in sensitivity over T/TD, that often uses a sensing moiety covalently attached to the channel itself, and that typically involves a T/TD-type blockade lifetime event with minimal or no internal blockade structure engineered [31,32]. The NTD approach, on the other hand, has significant improvement in versatility via use of non-covalently captured modulators that can be electrophoretically swapped out on a given channel by voltage reversal. The improvements in sensitivity derive from the measurable, non-trivial, stationary statistics of the channel blockades (and how this can be used to classify state with very high accuracy). The overall improvement in versatility is because all that needs to be redesigned for a different NTD experiment (or binding assay) is the linkage-interaction moiety portion of the bifunctional molecules involved. There is also the versatility that *mixtures* of different types of transducers can be used, a method that can’t be employed in single-channel devices that use covalently bound binding moieties (or that discriminate by dwell-time in the channel).

At the nanopore channel one can observe a sampling of bound/unbound states, each sample only held for the length of time necessary for a high accuracy classification. Or, one can hold and observe a single bound/unbound system and track its history of bound/unbound states or conformational states. The *single* molecule detection, thus, allows measurement of molecular characteristics that are obscured in ensemble-based measurements. Ensemble averages, for example, lose information about the true diversity of behavior of individual molecules. For complex biomolecules there is likely to be a tremendous diversity in behavior, and in many cases this diversity may be the basis for their function. The NTD Nanoscope may provide the means to ‘see’ individual biomolecular kinetics and dynamic behavior. There can also be a great deal of diversity via post-translational modifications such as with heterogeneous mixtures of protein glycoforms that typically occur in living organisms (e.g., for TSH and hemoglobin proteins in blood serum and red blood cells, respectively). The hemoglobin A1c glycoprotein, for example, is a disease diagnostic (diabetes), and for TSH, glycation is critical component in the TSH-based regulation of the endocrine axis. Glycation on antibodies can also be significant and nanopore detector work on blockade signals with multiple levels are described in [33]. Multi-component regulatory systems and their variations (often sources of disease) could also be studied much more directly using the NTD approach, as could multi-component (or multi-cofactor) enzyme systems. Glycoform assays, characterization of single-molecule conformational variants, and multi-component assays are significant capabilities to be developed further with the NTD approach, further details on NTD assaying will follow in a later section.

## Background

Background is first given on biophysics information flows that result in stationary statistics observations. Then background is provided on the use of stationary statistics signal processing in device enhancement and communication.

### Ponderable media flow phenomenology and related information phenomenology

An important part of the NTD methodology is to have a stable ion-channel *sized* such that it can be modulated by a *single*, non-translocating, molecule, where the channel is modulated such that it is significantly blockaded, and not at a fixed level -- e.g., where at least one of the transducer blockade states has multi-level structure (and not simply the approximately Gaussian noise profile of a fixed level blockade). Using a properly-sized channel, it is possible to *establish a modulated ion-flow*, through the alpha-hemolysin ion-channel for example, with a *single* NTD transducer molecule, where the NTD molecule is electrophoretically drawn into the channel.

Biology provides highly stable, nanometer-scale (0.1 -- 100 nm), protein-based ion-channels appropriately sized for the NTD methodology with single-molecule blockaders. A single molecule’s blockading interaction upon capture in an ion channel can be self-modulating upon capture (i.e., without a dominant interaction state), and this has been found in a number of experiments [34-46]. The mechanism of interaction involves transient chemical bond formation between transducer and protein, where each bound state between transducer and channel imprints on the surrounding ionic current flow to provide a fixed level which then transitions to a different fixed level upon the bond dissociation or transitions to a different bound state. Self-modulatory blockaders each have unique blockade signatures that can be resolved to very high confidence (with higher confidence the longer the observation time). Given the engineering freedom to design the self-modulatory molecules, and the generalizations in the standard periodic carrier based signal processing to stationary statistics carrier based signal processing, we arrive at a means to leverage ponderable media flow phenomena, and blockader interaction kinetics, into a stochastic carrier wave signal processing problem that can be solved using efficient dynamic programming table computational methods as described here.

A single molecule that is captured in an ion channel is not in its natural state, especially if made to modulate the channel (made to ‘dance’), e.g., to provide a multi-level (multi-state) modulation of the channel current. If the captured molecule is engineered (or selected) for use in transducing events, then the captured molecule will have at least two ion-channel blockade states. Given the freedom to adjust applied potential, pH, salt, and other factor so as to increase the number or distinctiveness of blockade states, highly sensitive biosensing arrangements can be established. Detector operation typically involves capturing and establishing flow modulators to produce a biosensing arrangement that responds to specifically designed, or selected, target stimuli (such as binding of the transducer molecule’s extra-channel binding moiety region to its target, see Fig. 1). The captured molecules are altered from their natural form, via stretching and conformational change, in the high electrophoretic force environment at the channel’s internal limiting aperture (that prevents the passage of dsDNA). As mentioned, the most unnatural aspect of a captured molecule, if an NTD modulator, is the unique channel blocking dance that is established upon capture. Multi-state capture configurations are possible because the captured molecules are brought into contact with the channel walls such that transient chemical interactions take place between portions of the captured molecule and portions of the channel wall, where chemical interactions are taken to include the following binding interactions, among others: electrostatic, pi-bond, ionic, polar covalent, dipole-dipole, hydrogen-bond, Van Der Waals bond, hydrophobic effect bond, and water-of-hydration effect bond.

In a general sense, the nanopore transduction detection method could be broadened to include a detection situation involving functionalization of any flow with stationary statistics, by use of flow modulators that have more than one stationary statistics phase of channel blockade, or flow modulation, where those phases are associated with states of the blockader. Anything that can be bathed in a stationary statistics flow can, thus, be observed by how it modulates that flow. This takes any widget and makes it a smart widget by allowing us to be better informed about its state, and state transitions. All that is required is establishing a stationary statistics flow that is modulated according to state of blockader in some desirable way.

For flow modulation at the nanometer-scale, it is important to note that we are at the scale of single molecules, for which the inherent coherence, and the time-frames for stationary statistics and clearly discernable states (statistical coherence), can be established for greater periods, allowing for greater statistical stability on the modulations produced by a single-molecule interaction (as with high precision atomic clocks being based on a single atom that use a periodic signal attribute in the standard EE signal processing manner).

The lipid bilayer is the main weakness in the protein-channel based approach. Device hardening may eventually be done using solid-state channels, or via hybrid protein channel & ‘soft’-state devices (via use of bilayer coat or scaffolding procedures).

Thus, a single molecule’s blockading interaction upon capture in an ion channel can be self-modulating upon capture (i.e., without a dominant interaction state), and this has been found in a number of experiments ([34-38] in particular). Self-modulatory blockaders each have unique blockade signatures that can be resolved to very high confidence over time. Given the engineering freedom to design the self-modulatory molecules, and the generalizations in the standard periodic carrier based signal processing to stationary statistics carrier based signal processing [47,48], we arrive at a means to leverage ponderable media flow phenomena, and interaction kinetics, into a stochastic carrier wave signal processing problem that can be solved by efficient dynamic programming table computational methods [49,50].

### Nanopore ion-flow based biosensing methods

**Fig. 2:**
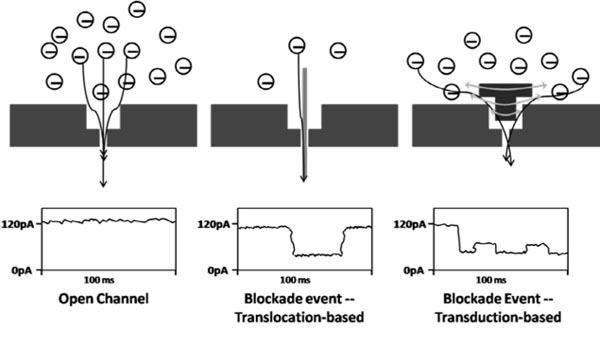
**Translocation information and transduction information.** (Left) Open channel. (Center) A channel blockade event with feature extraction that is typically dwell-time based. A single-molecule coulter counter. (Right) Single-molecule transduction detection is shown with a transduction molecule modulating current flow (typically switching between a few dominant levels of blockade, dwell time of the overall blockade is not typically a feature -- many blockade durations will not translocate in the time-scale of the experiment, for example, active ejection control is often involved).

**Fig. 3:**
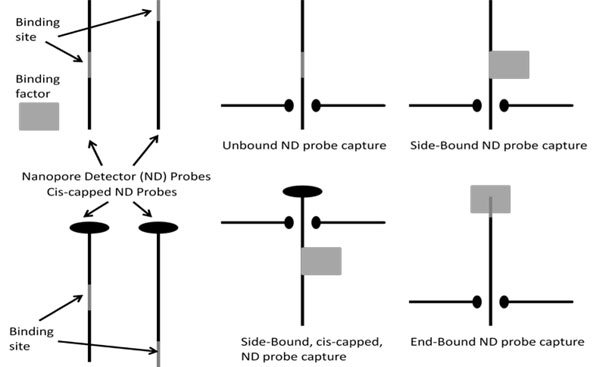
Nanopore Detector detection topologies involving polymer translocation or threading. The detection event is given by polymer (ssDNA in [7-26]) translocations that are delayed if bound (side and end configurations shown). If bound entity is on the trans side (with cis-side capped, or vice versa), and bound entity is a processive DNA enzyme, then sequencing may be possible as described in [15,27-30].

The standard Nanopore Detector (ND) detection paradigm, that is predominantly translocation (or dwell-time) based, is shown in Fig. 2 side-by-side with the Nanopore *transduction* detector paradigm. Figure 3 elaborates on the possible ND detection platform topologies possible with translocation-based approaches, where the difference in translocation times is often the critical information that is used. The difference in dwell times can depend on the off-binding time of the target binding entity (possibly in a high strain environment), where binding failure allows polymer (ssDNA) translocation to complete (and the channel blockade to end). By this mechanism, and its variants, bound probes can be distinguished from unbound. There are specificity limits on the melting-based detection, however, that are not a problem in the NTD approach.

## Methods and results

NTD-based applications and implementations are described for: (i) Biosensing; (ii) Assaying; and (iii) NTD Nanoscope Deployments for both device and unassembled kit platforms.

### NTD biosensing

NTD biosensing methods can involve a DNA modulator with linkages to an aptamer, antibody, or some other binding moiety, including simply a ssDNA overhang. The linkages needed to connect a DNA-based channel-modulator to a DNA-based aptamer involves a trivial join of the underlying ssDNA sequences involved. The linkage needed to connect a DNA-based channel-modulator to an antibody *could* involve use of linker technology, and this has been used in the past with dsDNA hairpins [33], but another, more commoditized route, easily accessible with use of the NADIR refined Y-shaped DNA channel modulators [36,44], is that the antibody need merely be tagged with the appropriate ssDNA strand, e.g., where the DNA sequence is complement to part of the ‘Y’ shaped DNA channel modulator, and antibody tagging with DNA is a standard service for use in immuno-PCR. Proof-of-Concept Experiments are described next for the streptavidin-biotin and DNA annealing model systems, a pathogen/SNP detection prototype, and for aptamer and antibody based detection.

### Model system based on streptavidin and biotin

A biotinylated DNA-hairpin has been engineered to generate two signals depending on whether or not a streptavidin molecule is bound to the biotin. In [6] we show transduction of bound/unbound signals at three different transducer concentrations and a range of binding target (streptavidin) concentrations. Rescaling is done on counts, with the count of events at the 0.05 µM Bt-8gC scaled up by a factor of 20, for example, for comparing event rate observations at different concentrations with the 1 µM Bt-8gC behavior (where a linear response with concentration will result in an overlay of the different plots according to class, as is seen over a two-magnitude range). Thousands of individual blockade observations are used to obtain the observation counts, and from this the apparent concentration at the various streptavidin concentrations.

### Model system based on DNA annealing

**Fig. 4:**
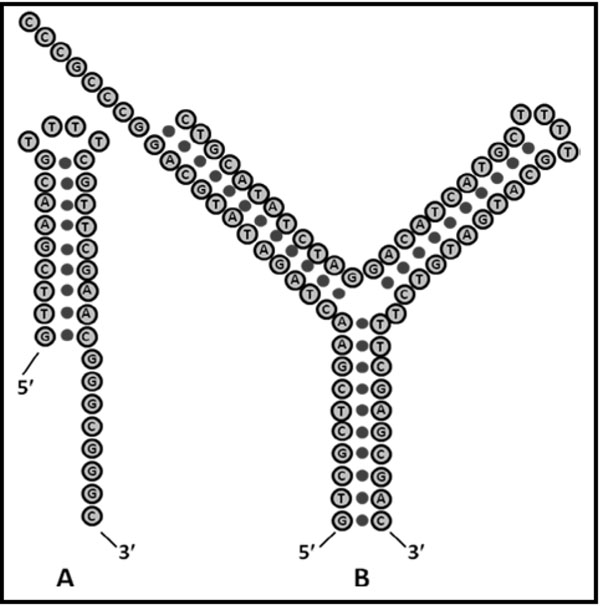
The Y-Anneal transducer, and its annealing target.

A unique, Y-shaped, NTD-aptamer is described in Fig. 4. In this experiment a stable modulator is established using a Y-shaped molecule, where one arm is loop terminated such that it can’t be captured in the channel, leaving one arm with a ssDNA extension for annealing to complement target.

A test of DNA annealing has been performed with the Y-shaped DNA transduction molecule indicated, where the molecule is engineered to have an eight-base overhang for annealing studies. A DNA hairpin with complementary 8 base overhang is used as the binding partner. In [6] we show the binding results at the population-level (where numerous single-molecule events are sampled and identified), where the effects of binding are discernible as are potential isoforms. The introduction of urea at 2.0 M concentration is easily tolerated (a mild chaotrope) and actually helps in discerning collective binding interactions such as with the DNA annealing.

### SNP detection efforts

A possible test of DNA SNP annealing is with the Y-shaped DNA transduction molecule shown in Fig. 4 (‘B’) that is minimally altered, and such that the SNP variant occurs in the Y-nexus region. For the case where digestion can’t conveniently provide extension only to one-side, a Y-shaped annealed dsDNA molecule can still be obtained, but such that the ssDNA extensions outside the annealed region are now free to extend on both arms of the Y-molecule.

**Fig. 5:**
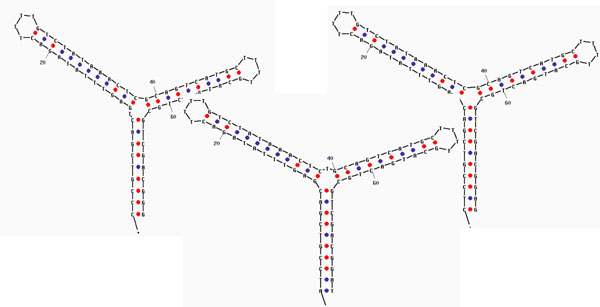
Shown are Y-shaped aptamers that have shown they have capture states with the desired blockaded toggling.

SNP variant detection is reduced to resolving the signals of two Y-shaped duplex DNA molecules, one with mismatch at SNP, one with Watson-Crick base-pairing match at SNP. In studies of Y-shaped DNA molecules, numerous Y-shaped DNA molecules were considered. Three variants that successfully demonstrated the easily discernible, modulatory, channel blockade signals are shown in Fig. 5[44]. In those variants we considered the Y-nexus with and without an extra base (that is not base-paired). If an extra base is inserted we explore the three positions at the Y (left and middle inserts shown in the left and center Y-molecules shown in Fig. 5.

The DNA molecular design used in [44] consists of a three-way DNA junction created: 5’-CTCCGTCGAC GAGTTTATAGAC TTTT GTCTATAAACTC GCAGTCATGC TTTT GCATGACTGC GTCGACGGAG-3’. Two of the junctions’ arms terminate in a 4T-loop and the remaining arm, of length 10 base-pairs, is usually designed to be blunt ended (sometimes shorter with an overhang). The blunt ended arm has to be carefully designed such that when it is captured by the nanopore it produces a toggling blockade. One of the arms of the Y-shaped aptamer (Y-aptamer) has a TATA sequence, and is meant to be a binding target for TBP. In general, any transcription factor binding site could be studied (or verified) in this manner. Similarly, transcription factor could be verified by such constructions, or the efficacy of a synthetic transcription factor could be examined.

In using the NADIR refinement process to arrive at the Y-transducer used in the DNA annealing test [44], we have demonstrated how *single-base insertions or modifications at the nexus of the Y-shaped molecule can have clearly discernible changes in channel-blockade signal*. Y-molecules as DNA probes with single point mutations discernible at the Y-nexus are explored in [44] (see Fig. 5). What is described in [44] is a linkage to a *na*nopore-detector *dir*ected (NADIR) search for aptamers that is based on bound-state lifetime measurements (or some other selection criterion of interest). NADIR complements and augments SELEX in usage. Further discussion of NTD Biosensing experiments along these lines is in the Discussion, for SNP, Aptamer, and Antibody based detection systems. Extensive further results in these areas are described in [51], as well.

### NTD assaying

Using a NTD Nanoscope, a single bound/unbound system can be held, observed, and its history of bound/unbound states can be tracked. The *single* molecule state-tracking with lengthy time averages allows measurement of molecular characteristics that are obscured in ensemble-based measurements. (The ensemble averages that underlie most approaches lose information about the true diversity of behavior of individual molecules.) For complex biomolecules there is likely to be a tremendous diversity in molecular behavior, and in many cases this diversity may be the basis for their function.

DNA termini are of critical importance for certain retroviral integrases and other biological processes – being able to study them, even comparatively, offers new avenues for understanding and drug selection (HIV integrase blockers). Information on the DNA molecules’ variation in structure and flexibility is important to understanding the dynamically enhanced (naturally selected) DNA complex formations that are found with strong affinities to other, specific, DNA and protein molecules. An important example of this is the HIV attack on cells. The DNA terminus properties of retroviral DNA molecules are found to exhibit greater flexibility than similar sequences, often marked by an increase in the number of blockade states, such as in the upper-level fine structure for the molecule terminating with GACG-3’ [42].

One of the most critical stages in HIV’s attack is the binding between viral and human DNA. The DNA molecule studied in this instance consists of the HIV consensus terminus at the end of the Y-aptamer arm – where it is exposed for binding to integrase. Since this molecule presents another blunt-ended dsDNA for capture, it is no surprise that such events occur. The signal analysis must separate between two classes of signal associated with these two dominant forms of capture -- associated with capture of the two blunt-ended DNA regions (at the base of the Y and at the end of the integrase-binding arm). With appropriate capture of the molecule at the base of the Y, this permits direct examination of protein binding to the terminal DNA region.

The NTD approach may provide the best means for examining other enzymes, and other complex biomolecules, particularly their activity in the presence of different co-factors. There are two ways that these studies can be performed: (i) the enzyme is linked to the channel transducer, such that the enzyme’s binding and conformational change activity may be directly observed and tracked or, (ii) the enzyme’s substrate may be linked to the channel transducer and observation of enzyme activity on that substrate may then be examined. Case (i) provides a means to perform DNA sequencing if the enzyme is a nuclease, such as lambda exonuclease. Case (ii) provides a means to do screening, for example, against HIV integrase activity (for drug discovery on HIV integrase inhibitors). Further details can be found in [45].

### NTD Nanoscope deployment: device & kit

#### The NTD single-molecule nanoscope device (fully-assembled)

A NTD Nanoscope for single molecule experiments is being developed by Meta Logos Inc. The starting point for the device deployment is a fully assembled bilayer workstation interfaced with a preconfigured computer for data acquisition and signal processing. Further details on the fully assembled deployment will not be discussed further in this paper.

#### The NTD Nanoscope kit device (unassembled)

A kit version of the NTD Nanoscope is possible where the components can be bought separately (e.g., vibration isolation table; patch clamp amplifier, etc.) for as little as $10,000 in total. Two elements are missing, however, in a practical deployment of this very new technology: (1) model test systems – in response to this Meta Logos is developing test buffers and test molecules with its kit along the lines of the successful experiments described in [34,36,38]; and (2) software with both local acquisition tools (see [38]) and off-site server or web-interfaced tool-sets (see [37]), the latter with more elaborate data analysis methods and services to be made available via Meta Logos server-linked signal processing via their kit or consulting arrangements. The NTD Kit Deployment involves the following:

(1) The NTD Device machined components and parts (from Meta Logos), and a shopping list of recommended commercially available components (vibration table, etc.), and chemicals.

(2) The NTD Set-up and Operation Manual (from Meta Logos).

(3) A configured NTD Kit Computer with local SSA software and meta-logos SSA server-link (from Meta Logos).

(4) A set of specialized buffer controls used for calibration (from Meta Logos).

(5) Selected model systems/controls (from Meta Logos, see Results)

(6) Data analysis and data repository services (from Meta Logos).

(7) Setup, calibration, and troubleshooting services (from Meta Logos).

The NTD Nanoscope is designed to be as self-calibrating as possible to provide enhanced, autonomous reliability: signals are normalized computationally with respect to physical parameters (e.g. temperature, ph, salt concentration, etc.), eliminating the need for complex (and expensive) physical feedback systems to stabilize the device. In what follows, further results are specifically provided involving (i) the Kit components; (ii) the NTD Model Systems; and (iii) the SSA Toolset & Server.

#### Kit components

**Fig. 6:**
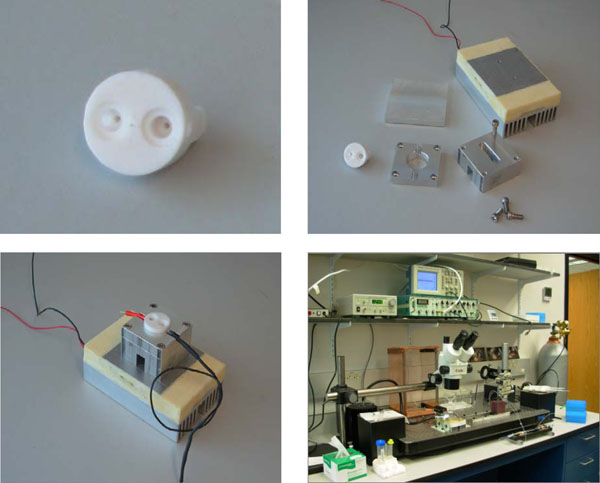
**The NTD Kit Components.** (Upper Left) One inch diameter Teflon core machined to contain two chambers that are connected by a segment of Teflon tubing. This tubing has one end open while the other is capped to restrict it’s opening to a tiny pin hole (Aperture) approximately 25 microns in diameter (not shown). (Upper Right) Aluminum square block is machined to support the core and provide access for illumination and electrode ports, and have easy assembly and mounting on the Peltier device. (Lower Left) The Teflon and aluminum components are shown seated on the Peltier device for temperature control of the wells. Silver chloride (Ag/AgCl) pellet electrodes are shown inserted into the cis and trans chambers port wells. (Lower Right) A NTD Nanoscope kit arrangement that uses a table-top vibration isolation table, has perfusion apparatus, Faraday cage, and linkage to a computer (to left of workstation, not shown).

In Fig. 6 is shown the NTD kit core Assembly with Peltier-based temperature controller model 350B manufactured by Newport Temperature. The A-M systems patch clamp amplifier model 2400 is used to impose a constant voltage across electrodes while monitoring picoampere fluctuations. A micro manipulator is used to deliver profusion tubing to *cis* chamber during channel acquisition. A TMC bench top vibration isolation table is used to support the core assembly and reduce vibration shock. With the NTD Kit we, thus, achieve a nanoscale single-molecule lab construction. This is obviously a low-cost method to perform nanoscale experiments. There is also a remarkable versatility in the incorporation of transducer, as will become more apparent in what follows.

#### Model systems

Specially engineered calibration probes have been designed to enable real-time self-calibration by generating a standard “carrier signal.” These probes can also be added to samples being analyzed to provide a run-by-run self-calibration. These redundant self-calibration capabilities result in a stable, user-friendly device that can be operated by an entry level lab technician. Similarly, model systems based on experiments described in [36,50] have been established to allow quick entre into various SNP-based and biosensing scenarios, including the following model systems:

(1) The streptavidin biosensor model system [6].

(2) The DNA annealing model system [44].

(3) The targeted pathogen model system [46].

(4) The SNP minority/majority population detection system [46].

(5) The aptamer-transduced detection of thrombin system [46,52]

(6) The antibody-transduced detection system using immuno-PCR style tagging with DNA, where complement is engineered to form a Y-transducer, or related, detection system [46].

#### SSA toolset & server

Although the nanopore transduction detector can be a self-contained device in a lab, external information can be used, for example, to update and broaden the operational information on control molecules (‘carrier references’). For the general NTD kit user, carrier reference signals and other systemically-engineered constructs can be used, for example, for a wide range of thin-client arrangements (where they typically have minimal local computational resources). The paradigm for both nanopore device and NTD kit implementations involve system-oriented interactions, where the kit implementation may operate on more of a data service/data repository level and thus need real-time (high bandwidth) system processing of data-service requests or data-analysis requests. Although not as system-dependent on database-server linkages, the more self-contained device implementation will still typically have, for example, local networked (parallelized) data-warehousing, and fast-access, for distributed processing speedup on real-time experimental operations.

For the general user and the NTD-Kit user, the server interfaces for the SSA Server facilities are being designed to provide services ranging from web-interface based services to an active, streaming, experimental calibration/troubleshooting effort. Nanopore Device and Nanopore Kit users will have the signal acquisition module, the tFSA, bundled with their hardware (on a preconfigured computer, see Table 2). This allows for bare-bones functionality with the bundling as a stand-alone device. When there is need for more refined signal processing, such as when using the weakness recovery protocol to acquire signal when the FSA fails, the SSA Server will have the full panoply of methods available to get a lock on the signal, and examine it further. The transfer of data at this juncture will also be efficiently pre-processed (e.g., the FSA acquisition will already be done) and will thereby allow for much lower bandwidth and latency in the networked signal processing. Biology has invented such structure repeatedly, such as with the retinal pre-processing in the human eye.

**Table 2:** Software Toolsets.

KIT-user on-site Software Toolset	General-user Web-Portal Toolset
FSA	FSA, FSA-tuning
HMM*	HMM, HMMD, etc.
SVM*	generalized SVM
	Tuning Metaheuristics
	NTD Calibration Services
	NTD Troubleshooting Servies
	CCC Shared Reference Signal Library

(small datasets only for onsite HMM and SVM, no distributed processing)	(large datasets for HMM and SVM training, with use of distributed processing and speciality hardware such as GPUs)

*specialized enhancement packages to be made available from Meta Logos in future refinements to their Nanoscope effort.

## Discussion

In what follows we describe efforts underway with NTD biosensing using Y-aptamers; NTD-based assaying; and for molecular capture for preprocessing and biofouling prevention.

### NTD Y-aptamer detection systems

#### SNP detection

**Fig. 7:**
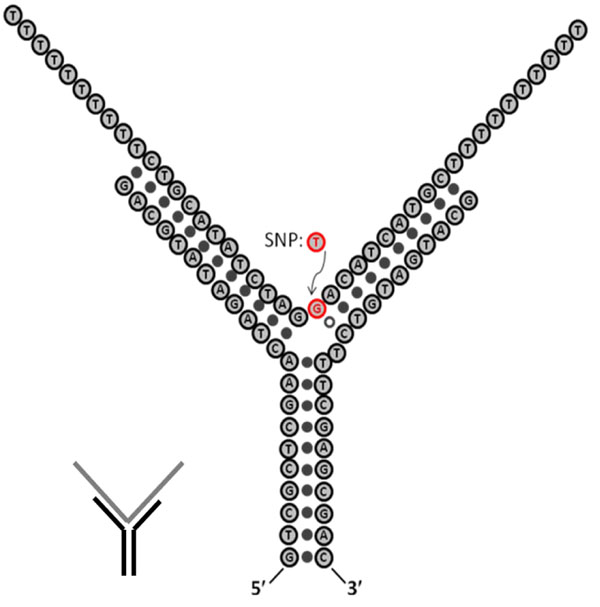
The Y-SNP with test complex is shown at the base-level specification and at the diagrammatic level, where a SNP base is as indicated. If the SNP is its variant form (typically only one other base possibility is common), then a base-pairing will not occur at the nexus of the Y-SNP shown (with the red base becoming a ‘T’ in the variant as indicated). This allows discrimination between the annealed forms with high accuracy, while also discerning from the signals produced by the non-annealed Y-SNP, where there is no target-bound, or only non-specific molecular interactions imparting much less conformational structure as occurs with the matching (or mostly matching) annealing interaction.

A test of DNA SNP annealing can be done with the Y-shaped DNA transduction molecule shown in Fig. 7, which is minimally altered (e.g., mostly common sequence identity) from the Y-annealing transducer studied in Fig. 4.

**Fig. 8:**
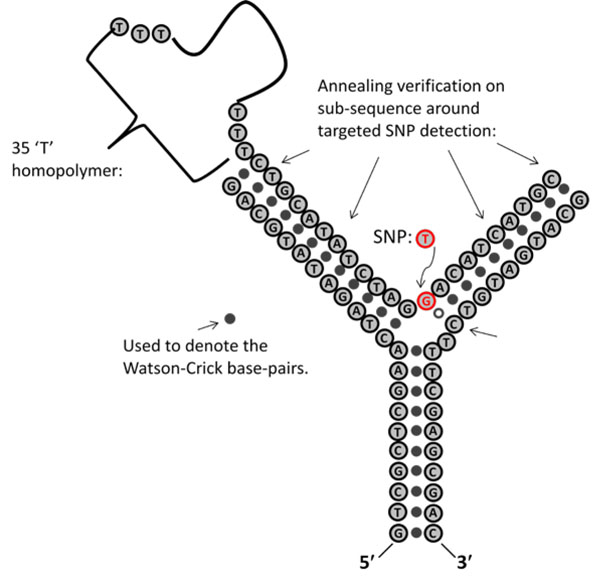
The Y-SNP test complex with 35 dT length overhang is shown at the base-level specification, where a SNP base is as shown. If the SNP is its variant form (typically only one other base possibility is common), then a base-pairing will not occur at the nexus of the Y-SNP shown. This allows discrimination between the annealed forms with high accuracy, while also discerning from the signals produced by the non-annealed Y-SNP, where there is no target-bound, or only non-specific molecular interactions imparting much less conformational structure as occurs with the matching (or mostly matching) annealing interaction.

Once the Y-SNP transducer has been tested on a single-species of short overhang length test molecules the next experimental challenge will be to detect SNP variants using the Y-SNP transducer probe in the presence of a heterogeneous length mixture (some with target SNP region of interest), with overhang as shown in Figure 8.

The value of 35 ‘T’s on the extension is to also match the approximate extension, with the same ‘Y’-sequence (except for a 4 dT cap) as the previously blunt-ended annealed conformation. SNP variant detection is reduced to resolving the signals of two Y-shaped duplex DNA molecules, one with mismatch at SNP, one with Watson-Crick base-pairing match at SNP. From the above, it is clear that the NTD method provides a viable prospect for SNP variant detection to very high accuracy (possibly the accuracy with which the NTD can discern DNA control hairpins that only differ in terminal base-pair). SNP detection via *translocation-based* methods, on the other hand, must discern between two SNP variants according to the different dwell times of the complement-template annealed SNPs, until dissociation from the template allows translocation of the blockading dsDNA annealed conformation.

#### Aptamer-based detection

**Fig. 9:**
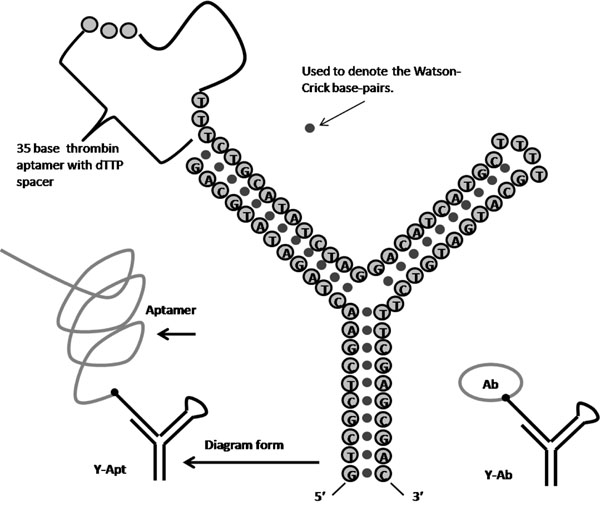
The thrombin aptamer from [52] is 5’-CACTGGTAGGTTGGTGTGGTTGGGGCCAGTG-3’.

Aptamers are especially appropriate for study by nanopore detection due to the fact they can be designed with an end to be captured by and modulate a nanopore (i.e., the captured end is dsDNA), while other parts of the aptamer are designed to bind a specific target. This directly provides a NTD transducer if one or both of the bound/unbound states (captured in the channel at the dsDNA end) provides distinctive channel modulations. The binding statistics derived from the study of aptamers in a nanopore detector can also be used in the design of the aptamer itself, e.g., NADIR selection instead of further SELEX-based selection [44]. In Fig. 9 we see the first aptamer test case to be considered, where we seek to detect thrombin [52] in one case, and IgG [53] in another. In one effort, we use the thrombin aptamer found by Ikebukuro et al [52], which is selected via SELEX and EMA and is a 31-mer,that we link by a 4 dT spacer to the Y-transducer (see Fig. 9).

#### Antibody-based detection

Linkage of ssDNA to antibody is commonly done in immuno-PCR preparations, so another path with rapid deployment is to make use of a linkage technology that is already commoditized, e.g., the molecules required for the antibody-based biosensing with this approach are simple (non-specialty) molecular components. The core issue to be resolved here is whether a good NTD signal can be produced with immuno-PCR tagged antibodies that are designed to anneal to another DNA molecule to form an NTD ‘Y-transducer’ (see Fig. 9, lower right). From previous efforts [33], with more complicated EDC linkages between a modified thymine and an antibody, it is clear that there are strong prospects for success with this method. What is sought is not just further validation of the method, however, but a less expensive, accessible, platform from which to refine and develop NTD-based systems.

### NTD based assaying systems

Part of the complexity of glycoforms, and other modifications, of proteins such as hemoglobin and TSH, is that these glycoforms are present as a heterogeneous mixture, and it is the relative populations of the different glycoforms that may relate to clinical diagnosis or identification of disease (such as prion exposure [54]). To this end, a protein’s heterogeneous mixture of glycations and other modified forms might be directly observable with a NTD nanoscope, and this constitutes the clinically relevant data of interest in many situations, not simply the concentration of some particular glycoform. Furthermore, it is the transient, dynamic, changes of the glycoform profile that is often the data of interest, such that a real-time profile of glycoform populations may be of clinical relevance, and obtaining such real-time profiling of modified forms (glycoforms, etc.) would be another area of natural advantage for the NTD approach.

NTD assaying provides a new technology for characterization of transient glycoprotein complexes, antibody assays, and multicomponent enzyme assays. For further discussion see [46].

### Molecular capture & TERISA

It is possible to couple NTD methods with antibody capture systems, or any specific-binding capture system (e.g., MIP-capture, DNA microarray, or aptamer-based capture systems could be used as well, for example). This allows for a pre-processing step that can concentrate the analyte of interest in a capture matrix, as well as provide a means to eliminate biofouling components from entering the nanopore detector and possibly damaging the bilayer [46] (biofouling is a serious complication in solid state nanopore efforts).

Antibody capture in the TERISA approach [46] allows an NTD experiment to report on the presence of the target molecules via indirect observation of transduction molecule signal corresponding to UV cleaved NTD substrate molecules (that are freed from the capture matrix). Commercially produced systems are available with matrices pre-loaded with immobilized Fc-binding antibodies; the secondary antibody can then be introduced and bound by the Fc-binding Ab’s to establish the desired, immobilized, specific-binding matrix (analogous to sandwich-ELISA). If solution with target molecule is now repeatedly washed across the immunosorbant surface, an immobilized concentration of that target molecule can be obtained. We can now introduce our primary antibody that targets the immobilized antigen (‘sandwiching’ it).

The further novel aspect of this setup is to now have the primary antibody linked to an enzyme that acts on a NTD transducer substrate (analogous to a fluorescent substrate in ELISA). By taking some of the methodology from the ELISA approach (enzyme-linked immunosorbent assay), and merging it with unique aspects of the nanopore detection approach, we have the ‘Transducer Enzyme-Release with ImmunoAbsorbent Assay’ (TERISA) approach, [55], where “Sandwich TERISA” is assumed to typically be the case, since specific immobilization is desired.

Analogous to real-time PCR, where a qualitative PCR result is self-calibrated according to real-time values to obtain quantitative PCR results, we can do the same with the TERISA and TARISA biosensing methods outlined here. In other words, for all three methods with real-time observation (RT-TARISA, RT-TERISA, E-phi Contrast RT-TERISA), we can shift to a more quantitative footing (as with RT-PCR or RT-ELISA). In our case this is trivially achieved, since the data-acquisition and signal processing is already in use and operating in real-time. This real-time tracking information helps to stabilize the method and complements the biosensing capability with a self-calibrating quantitative assaying capability (where highly accurate resolution of mixtures of DNA hairpin molecules are shown to be possible [38]). See [46] for other applications of note, such as: NTD operation as a Gel assay; NTD use as a

Nanopore Processing Unit (NPU); NTD use in Pathogen Detection; and NTD-based stationary statistics based DNA sequencing approach.

## Conclusion

NTD methods offer prospects for improved SNP detection and improved detection of other localized DNA regions in general, with a profound impact on how SNP detections are done in diagnostics. NTD methods are shown to offer significant advantage in the aptamer and antibody based biosensing systems as well. Currently there are a huge variety of tests for various forms of cancer or genetic disorders, for example, and the NTD approach offers a highly-accurate, inexpensive, and fast test result on a platform functionalized for all tests – where the tests may be done simultaneously on the same sample using a mixture of the appropriate NTD probes.

Nanopore transduction detection provides a highly discriminative method for biosensing, assaying and performing medical diagnostics using highly specific binding to some biomarker (e.g., antibody-based binding or aptamer-based binding). Kidney damage, for example, is often undiagnosed until significant damage has already occurred and preventive action is limited [56-59]. It is hypothesized that NTD probes can also be developed for early stage kidney disease detection and other disease detection, via biomarker biosensing with high sensitivity, without loss of necessary specificity in the standard clinical tests of the future. The overall market impacted by this technology is very broad and includes the diagnostics, pharmaceutical, and biotechnology industries.

We describe NTD Nanoscope Device deployments that provide an inexpensive, quick, accurate and versatile method for biosensing, assaying and performing medical diagnostics. We also describe the model systems examined on the NTD Nanoscope. The NTD approach provides a significant new tool for biology, biotechnology, public health and biodefense.

## General methods

### Experimental setup

Each experiment described in what follows was conducted using one alpha-hemolysin channel inserted into a diphytanoyl-phosphatidylcholine/hexadecane bilayer, where the bilayer was formed across a 20-micron diameter horizontal Teflon aperture [34]. The bilayer separates two seventy-microliter chambers containing 1.0 M KCl buffered at pH 8.0 (10 mM HEPES/KOH). A completed bilayer between the chambers was indicated by the lack of ionic current flow when a voltage was applied across the bilayer (using Ag-AgCl electrodes). Once the bilayer was in place, a dilute solution of α–hemolysin (monomer) was added to the *cis* chamber. Self-assembly of the α–hemolysin heptamer and insertion into the bilayer results in a stable, highly reproducible, nanometer-scale channel with a steady current of 120 pA under an applied potential of 120 mV at 23°C (± 0.1 °C using a Peltier device). Once one channel formed, further pores were prevented from forming by thoroughly perfusing the *cis* chamber with buffer. Molecular blockade signals were then observed by mixing analytes into the *cis* chamber. The experiment is performed on a vibration isolation table to keep the bilayer from rupturing (and ending the experimental setup). As mentioned earlier, the bilayer is the main weakness in the device operability, and it is primarily due to the problem of bilayer rupture, although permeability is another complicating factor in some circumstances as well. These are ongoing areas of research and refinements to the methods along these lines will not be described in this paper.

### Channel current cheminformatics architecture

A protocol has been developed for the discovery, characterization, and classification of localizable, approximately-stationary, statistical signal structures in stochastic sequential data, such as channel current data [46].

The channel current cheminformatics (CCC) pattern recognition informed signal processing architecture is described in [37,38,40,60]. The processing is designed to rapidly extract useful information from noisy blockade signals using feature extraction protocols, wavelet analysis, Hidden Markov Models (HMMs) and Support Vector Machines (SVMs). For blockade signal acquisition and simple, time-domain, feature-extraction, a Finite State Automaton (FSA) approach is used [61] that is based on tuning a variety of threshold parameters. A generic HMM is then used to characterize current blockades by identifying a sequence of sub-blockades as a sequence of state emissions [38,62,63]. The parameters of the generic-HMM can then be estimated using a method called Expectation/Maximization, or EM [62], to effect de-noising. The HMM method with EM is part of the standard implementation used in what follows. Classification of feature vectors obtained by the HMM for each individual blockade event is then done using SVMs. For the nanopore detector augmented with auxiliary molecules, much more data is usually needed to properly train the Machine Learning algorithms. The distributed training of these algorithms (recently established in [64]) is a critical component in real-time signal processing [40,60]. The CCC software helps in the discovery, characterization, and classification of localizable, approximately-stationary, statistical signal structures in channel current data, and changes between such structures. Along the lines of previous work in channel current cheminformatics [34,35,37-39,63], the CCC real-time implementation is used for analysis of the data to be measured, and refined as needed.

### The SSA Server

The SSA Protocol entails methods for signal acquisition, feature extraction, classification and clustering (see [34,37,38] for details). For Nanoscope Device or Nanoscope Kit users, a further refinement in the standard web-interfaces to SSA Web-Server methods [37] is to provide a streaming-data SSA-Server access. The latter setting offers Nanoscope calibration and troubleshooting capabilities in operational settings, as well as access to distributed real-time speedup on core computational methods. Thus, the entire pipeline of signal processing needed to go from raw streaming data to concise results can be passed to the SSA-Server. Usually it is more efficient, however, to separate the acquisition module (the tFSA) and bundle that with the device (or kit) to enable a bare-bones operational capability locally for signal acquisition, then enable feature extraction and classification/clustering enhancements via centralized maintenance of an SSA-Server algorithm toolset and data library. Many of the latter analyses can be done in real-time, for the experiments shown in the Methods for example, where a networked device is already set up as described. This same deployment is, thus, also sought in the Nanoscope Device or Nanoscope Kit development and is thereby already validated with the results on the existing system over the past several years. What this means for the signal processing needs is that the latency of a large raw data transfer is largely eliminated by the local pre-processing to perform rudimentary signal acquisition, which only passes acquired signal onto the network. For gene finding in mammals, for example, the gene regions amount to about 3% of the genome, so crude recognition of such would reduce acquisition and transmission of data by a factor of 33. The drop in bandwidth needs in NTD channel current signal processing can be even more pronounced.

## Competing interests

The authors declare that they have no competing interests.
